# Microwave & magnetic proteomics of macrophages from patients with HIV-associated cognitive impairment

**DOI:** 10.1371/journal.pone.0181779

**Published:** 2017-07-26

**Authors:** Yisel M. Cantres-Rosario, Frances M. Acevedo-Mariani, Juliana Pérez-Laspiur, William E. Haskins, Marines Plaud, Yadira M. Cantres-Rosario, Richard Skolasky, Israel Méndez-Bermúdez, Valerie Wojna, Loyda M. Meléndez

**Affiliations:** 1 Department of Microbiology and Medical Zoology, University of Puerto Rico, Medical Sciences Campus, San Juan, Puerto Rico; 2 Department of Chemistry, University of Puerto Rico, Rio Piedras Campus, San Juan, Puerto Rico; 3 RCMI Translational Proteomics Center, University of Puerto Rico Medical Sciences Campus, San Juan, Puerto Rico; 4 University of Texas, San Antonio, Texas, United States of America; 5 John Hopkins University, Department of Orthopedic Surgery, Baltimore, Maryland, United States of America; 6 Department of Biostatistics and Epidemiology, University of Puerto Rico Medical Sciences Campus, San Juan, Puerto Rico; 7 Department of Medicine, Neurology Division, University of Puerto Rico Medical Sciences Campus, San Juan, Puerto Rico; Imperial College London, UNITED KINGDOM

## Abstract

**Objective:**

HIV-infected monocytes can infiltrate the blood brain barrier as differentiated macrophages to the central nervous system, becoming the primary source of viral and cellular neurotoxins. The final outcome is HIV-associated cognitive impairment (HACI), which remain prevalent today, possibly due to the longer life-span of the patients treated with combined anti-retroviral therapy. Our main goal was to characterize the proteome of monocyte-derived macrophages (MDM) from HACI patients, and its association with their cognitive status, to find novel targets for therapy.

**Methods:**

MDM were isolated from the peripheral blood of 14 HIV-seropositive women characterized for neurocognitive function, including: four normal cognition (NC), five asymptomatic (A), and five with cognitive impaired (CI). Proteins from macrophage lysates were isobaric-labeled with the microwave and magnetic (M2) sample preparation method followed by liquid chromatography-tandem mass spectrometry-based protein identification and quantification. Differences in protein abundance across groups classified by HACI status were determined using analysis of variance.

**Results:**

A total of 2,519 proteins were identified with 2 or more peptides and 28 proteins were quantified as differentially expressed. Statistical analysis revealed increased abundance of 17 proteins in patients with HACI (p<0.05), including several enzymes associated to the glucose metabolism. Western blot confirmed increased expression of 6-Phosphogluconate dehydrogenase and L-Plastin in A and CI patients over NC and HIV seronegatives.

**Conclusions:**

This is the first quantitative proteomics study exploring the changes in protein abundance of macrophages isolated from patients with HACI. Further studies are warranted to determine if these proteins may be target candidates for therapy development against HACI.

## Introduction

The human immunodeficiency virus type 1 (HIV) pandemic has caused more than 35 million deaths since its discovery [1]. HIV infiltrates to the central nervous system (CNS) carried mostly by activated monocytes crossing the blood brain barrier (BBB) early in the infection. Once in the CNS, HIV-infected monocytes mature into macrophages and secrete cellular and viral toxins that induce neuronal damage, promoting the activation of local brain cells and persistent inflammation [2]. The macrophages also become major viral reservoirs in the CNS [3]. The inflammation and secretion of soluble neurotoxic factors by HIV-infected and non-infected activated cells trigger neuronal dysfunction. Together, these are the processes underlying the development of HACI. Symptomatology ranges from asymptomatic to full blown dementia. In the era of cART [4,5], approximately 10 to 50% of the individuals infected with HIV still develop a mild form of neurocognitive impairment, highlighting the urgent need to find novel targets for therapy development against HACI.

Since monocytes and macrophages are the key players in HACI, understanding the proteins and pathways altered in macrophages upon HIV infection, might help in preventing this disease. One of the neurotoxic proteins secreted by HIV-infected MDM and microglia is cathepsin B [6–8], a lysosomal cysteine protease. HIV infection disrupts cathepsin B interaction with its natural inhibitors in macrophages: cystatins B and C [6,9,10]. Moreover, cathepsin B and cystatin B are increased in monocytes from HIV-infected women with CI compared to HIV-seropositive women with normal cognition [7]. These results suggest that the uncontrolled expression and secretion of these proteins might be possible actors in the development of HACI.

Several proteomics approaches have been applied in the search for better drugs for HACI patients. These include surface enhanced laser desorption/ionization (SELDI)-time of flight (TOF) [4,11], stable isotope labeling of cells in culture (SILAC) [12], two-dimensional difference in gel electrophoresis (2-DIGE) [13], and isobaric tag for relative and absolute quantification (iTRAQ) of MDM secretome [14]. Although several *ex vivo* studies have investigated the changes in monocyte proteome from HIV-infected patients using these diverse proteomics approaches, none of these methods have been applied to identify macrophage intracellular proteins from HACI patients with cART. It is the macrophage, the cell releasing virus particles and toxins to the CNS. In our study, we selected macrophages after isolation and differentiation of blood monocytes from patients with HACI *in vitro*, to determine the relation between the changes in MDM proteome with their degree of neurocognitive impairment. To answer this question, we applied a rapid quantitative proteomics approach involving isobaric labeling with the microwave and magnetic (M2) proteomics sample preparation method [15–18] followed by liquid chromatography-tandem mass spectrometry (LC/MS/MS)-based protein identification and quantification. This method revealed differentially expressed proteins in MDM from 14 HIV-seropositive patients: four with normal cognition (NC), and 10 with HACI: five asymptomatic (A), and five neurocognitive impaired (CI). Seventeen proteins were increased in patients with HACI. One of these proteins, L-plastin, was confirmed by Western blot and four proteins (fructose-bisphosphate aldolase, glyceraldehyde-3-phosphate dehydrogenase, phosphoglycerate kinase, and pyruvate kinase), related to glucose metabolism approached significance during validation. Our results provide important information for additional protein targets related to HACI that deserve further studies in a higher number of patients.

## Materials and methods

### Patients

The study patients are from the Hispanic-Latino longitudinal cohort of HIV-seropositive women, followed since 2001 as part of the Specialized Neuroscience Research Program at the University of Puerto Rico, Medical Sciences Campus. The study was focused in women because this cohort was originally funded for two projects, one requiring women for hormone studies. Samples for this cross-sectional study were collected from 2009–2011 with funding from NIMH and approval of the Institutional Review Board (UPR-MSC; IRB# 0720109), Human Research Subjects Protection Office. The patients signed an informed consent for this study. Patients with hepatitis C, positive toxicology, or any other neuro-infectious disease were excluded. The neuropsychological performance was determined using Memorial Sloan Kettering (MSK) rating scale as modified by Marder et. al (2003) [19] stratifying patients in the following categories: normal cognition (NC) with MSK of zero, Asymptomatic (A) with MSK of 0.5, and cognitive impaired (CI) with MSK ≥ 1. All patients were evaluated with neuropsychological tests, macroneurological exam, and activities of daily living as previously described in Wojna et al (2006) [20].

### Cell cultures

Blood samples were collected from all patients in the mornings, prior to Neurological and Neuropsychological testing. Peripheral blood mononuclear cells (PBMCs) were isolated from the peripheral blood (40 mL) of patients using Lymphosep^®^ medium and gradient centrifugation (MP Biomedicals, Solon, Ohio) of PBMCs. All the PBMCs isolation and cultures were conducted by the same technician. Cells were cultured in RPMI supplemented with 20% heat-inactivated FBS (Sigma-Aldrich, St. Louis, MO), 10% heat-inactivated human serum (Sigma-Aldrich), and 1% Pen/Strep (Sigma-Aldrich) in T-25 flasks at a concentration of 1.5×10^6^cells/mL. MDM were selected by adherence after 7 days in culture. Half of the medium was changed every 3 days for all cultures. On day 6, media was replaced with serum-free media, and cells were collected 24 hours later following removal of serum-free media and preparation of cell lysates.

### Specimens from study patients

Patient specimens were stratified into 4 isobaric-labeled pools in a manner similar to that previously described in other M2 proteomics studies [15–18]: four patients with normal cognition (MSK = 0) with tandem mass tag (TMT) labels 126–129 as pool 1; five asymptomatic patients (MSK = 0.5) with labels 126–130 as pool 2, and five CI patients (MSK = 1) with labels 126–130 as pool 3. Label 131 combined the Pool 1, 2, and 3 as reference pooled material made by adding the same protein amount from all specimens (NC, A, and CI).

### Preparation of cell lysates

Lysis buffer (100μL of 5mM Tris-HCl buffer at H 8.0, 0.1M Triton X-100 and protease inhibitor cocktail) was added to macrophages for 30 minutes on ice and detached with a cell scrapper. Cell lysates were vortexed and centrifuged at 4°C for 10 minutes at 1,500 rpm. Supernatants were collected and stored in aliquots at -80°C. Protein concentration was determined using the BCA assay (Bio-Rad, Hercules, California).

### M2 proteomics sample preparation

Cell lysates (100μg) were dried in Speed Vacuum and then reconstituted with 100μL of Equilibration Buffer (200mM NaCl) (Acros Organics, Geel, Belgium), 0.1% Trifluroacetic acid (TFA; Sigma-Aldrich) in HPLC water (JT Baker, Center Valley, PA). The C8 magnetic BCMag beads (BioClone, San Diego, CA) were mixed with 50% Methanol (Fisher Optima, Waltham, MA) at a concentration of 50mg/ml. After reconstitution, 10μL of magnetic beads were transferred (50mg/ml) to a micro-centrifuge tube and placed on magnet for three minutes. After removal of supernatants, 100μL of equilibration buffer was added and washed by centrifugation. The supernatant was removed and the procedure was repeated three times for three minutes each. A total of 100μL of sample was mixed with 1/3^rd^ volume of binding buffer (800mM NaCl in 0.4% TFA) and added to the beads in the micro-centrifuge tube. The sample and beads were mixed again and after 5 minutes at RT, the tubes were placed on the magnet and the supernatant was removed. A volume of 150μL Triethyl Ammonium Bicarbonate (TEAB) buffer (Sigma-Aldrich) was added and placed again on magnet for three minutes and the supernatant was discarded. This was repeated three times. A volume of 150μL of 10mM of Dithiothreitol (DTT) (Agilent, Santa Clara, CA) was added and the tube was incubated for 10 seconds in the microwave. The DTT was removed by placing on the tube in the magnet for three minutes. A volume of 150μL of 50mM of Iodoacetamide (IAA) (GE Healthcare, Little Chalfont, UK) was added and the tube was microwaved for 10 seconds. The IAA was discarded after placing the tube on magnet for three minutes. The TEAB buffer (150μL) was added and the tube placed on the magnet for three minutes and the supernatant was discarded. This was repeated three times. A total of 100μL of enzyme solution, at ratio 1:25 trypsin: protein (Thermo Scientific; in 40mM TEAB buffer) was added to the beads and microwaved for 60 seconds. The tube was placed again on the magnet for three minutes and the supernatant was carefully eluted into new tubes, on ice. The samples were stored in -80°C until TMT labeling.

### Isobaric labeling

Immediately before use, the 6-plex TMT reagents (Thermo Scientific 126-131Da) were equilibrated to room temperature. For the 0.8 mg vials, 41μl of anhydrous acetonitrile (Thermo Scientific) was added to each tube and dissolved for 5 minutes with occasional vortex and incubated in the microwave for sixty seconds (triplicate of twenty seconds each). Thereafter, 8μl of 5% hydroxylamine (Sigma-Aldrich) in 1 M TEAB solution was added to the sample and incubated for 15 minutes to quench the reaction. Samples were combined at equal amounts and stored at -80°C.

### LC/MS/MS with protein database searching

LC/MS/MS was performed as previously described [2] by the RCMI Protein Biomarker Core, University of Texas, San Antonio, Texas. Briefly, LC/MS/MS was performed with a split-less nanoLC-2D pump (Eksigent, Livermore, CA, USA), a 50-μm id column packed with 7 cm of 3 μm-od C18 particles, and a hybrid linear ion trap-fourier-transform tandem mass spectrometer (LTQ-ELITE; Thermo Fisher, San Jose, CA, USA) operated with a lock mass for calibration. For unbiased analyses, the top six most abundant eluting ions were fragmented by data-dependent high-energy collision-induced dissociation. The reverse-phase gradient was 2 to 62% of 0.1% formic acid in acetonitrile over 60 min at 350 nL/min. All MS/MS spectra from tryptic peptides were analyzed using the probability-based protein database-searching algorithm Mascot (Matrix Science, London, UK; version 2.4.1). The SwissProt_041614 protein database (Homo sapiens; 20,340 sequences) was employed with a product ion mass tolerance of 0.050 Da and a precursor ion tolerance of 10.0 ppm. A static carbamidomethyl modification was selected for cysteine residues, while oxidation of methionine residues, N-terminal pyroglutamate and N-terminal acetylation were selected as variable modifications. Scaffold (version Scaffold_4.6.1, Proteome Software Inc., Portland, OR) was used to confirm MS/MS- based assignments. Peptides were accepted if they could be established at greater than 80.0% probability by the Peptide Prophet algorithm [21] with Scaffold delta-mass correction. Peptide false discovery rate was 1.0%. Protein assignments were accepted if they could be established at greater than 90.0% probability and contained at least 2 identified unique peptides. Protein probabilities were assigned by the Protein Prophet algorithm [22]. Proteins that contained similar peptides and could not be differentiated based on MS/MS analysis alone were grouped to satisfy the principles of parsimony. Proteins sharing significant peptide evidence were grouped into clusters. The protein false discovery rate was 6.7%.

### Ingenuity pathway analysis

From the 28 proteins identified, the significant differentially expressed proteins (n = 17) were imported to IPA software using their Swiss-protein accession ID and the fold change of the TMT labeling relative intensities to perform a core analysis on each of the following comparisons: A vs. NC, CI vs. NC and CI vs. A. IPA software linked the proteins to the relevant canonical signaling pathways, diseases and disorders, molecular functions and a predicted network of interactions.

### Western blotting

Fourteen of the 17 differentially expressed proteins were investigated by western blot. Western blot verification was conducted with the same samples used for TMT labeling. Briefly, 20μg of protein from each cell lysate was separated by SDS-PAGE, fixed, and electro-transferred to PVDF Membranes (BioRad). Membranes were incubated overnight at 4°C with primary antibodies including: mouse anti-actinin alpha 1 (1:1,000; R&D systems, Minneapolis, MN), mouse anti-aldolase (1:1,000; Abcam, Cambridge, United Kingdom), mouse anti-β-Actin (1:5,000; Sigma-Aldrich), mouse anti-cathepsin B (1:250; Sigma-Aldrich), mouse anti-filamin A (1:1,000; Abcam), mouse anti-galectin-3 (1:250; Abcam), mouse anti-heat shock protein (HSP70) (1:2,5000; R&D), mouse anti-moesin (1:1,000; Abcam), rabbit anti-6-phosphogluconate dehydrogenase (PGD) (1:5,000; Abcam), mouse anti-phosphoglycerate kinase-1 (PGK1) (1:1,000; Abcam), rabbit anti-L-Plastin (1:10,000; Abcam), mouse anti-tubulin-alpha (1:1,000; Thermo-Scientific), mouse anti-GAPDH (1:1,000; Santa Cruz Biotechnologies, Dallas, TX) or mouse anti-Vimentin (1:1,000; Abcam). Membranes were washed and incubated with horseradish peroxidase (HRP)-conjugated rabbit anti-mouse (1:10,000) or goat anti-rabbit (1: 10,000) secondary antibodies (Sigma-Aldrich). HRP activity was visualized by an enhanced chemiluminiscence detection procedure (Thermo Scientific). The band volume intensity from each protein was measured using ImageLab software (Bio-Rad), and normalized against the band volume of GAPDH for each PVDF membrane.

### Statistics

Mean and standard deviation of relative intensities of the identified proteins by cognitive group (NC; A; and CI) were calculated. Analysis of variance was used to determine the presence of between group differences in relative abundance. For proteins with significant between group differences in relative abundance, post-hoc pairwise comparison using Student’s t-test was used. Thresholds for statistical significance were *p<0.05 and **p<0.01, while the threshold for practical significance was a fold-change (F.C.) of 1.5. SAS software version 9.3 (SAS Institute, Cary NC) was used for all analyses. The western blots were analyzed using One Way Analysis of Variance (ANOVA), with Tukey’s post-hoc tests. Same thresholds described before for statistical significance were maintained. Tests were performed using GraphPad Prism 6.0 software (San Diego, CA).

## Results

### Isobaric labeling of proteins in specimens from study patients

The HACI classification of the 14 Hispanic Latino women that provided specimens for M2 proteomics analysis is described in Table 1. Ten of the 14 patients were taking cART. We did not find significant differences in CD4 count, plasma viral load or CSF penetration index (CPE) between HIV seropositive subjects with normal cognition and those with HACI (p>0.05) by ANOVA. The viral load of 1.7 log 10 = 50 copies/mL present in 7/14 or 50% of the total patients indicates that these patients are virally suppressed. Viral suppression by HACI category was: 25% (1/4) for NC; 60% (3/5) for A; and 60% (3/5) for CI. Table 1 also shows the patient population stratified into 3 label pools that included: four patients with normal cognition (MSK = 0) with TMT labels 126–129 as pool 1; five asymptomatic patients (MSK = 0.5) with TMT labels 126–130 as pool 2, and five CI patients (MSK = 1) with TMT labels 126–130 as pool 3. TMT label 131 combined the Pool 1, 2, and 3 as reference pooled material made by adding the same protein amount from all specimens (NC, A, and CI).

**Table 1:** Patient samples used for TMT labeling.

Patient Number	Visit	MSKN	HACI category	TMT Label*	Pool	Age	Plasma Viral Load (Log_10_ copies/mL)	CD4 count (cells/mm^3^)	CPE^a^	Combined Antiretroviral Therapy (cART)
59	13	0	NC	126	1	41	1.7	506	2.5	Combivir, Efavirenz, MTC, Folic Acid
119	5	0	NC	127	1	32	5.0	621	1	Ritonavir, Saquinavir, Truvada, MTV, Folic Acid
128	5	0	NC	128	1	31	3.7	579	1.5	Combivir, Nelfinavir
19	15	0	NC	129	1	47	2.7	356	2.5	Reyataz, Epzicom, MTV
180	1	0.5	A	126	2	42	1.7	563	2	Didanosine
181	1	0.5	A	127	2	53	1.7	520	7	Combivir, Nelfinavir
56	11	0.5	A	128	2	48	2.7	372	0	No
106	5	0.5	A	129	2	37	3.7	456	0	No
168	1	0.5	A	130	2	44	1.7	36	0	No
179	1	1	CI	126	3	52	1.7	916	2	Kaletra, Septra, Truvada, MTV, Folic Acid
172	2	1	CI	127	3	48	1.7	262	6	Enfuvirtide, Raltegravir, Etravirine
166	5	1	CI	128	3	63	N/A	N/A	10	Nelfinavir, Trizivir
40	13	1	CI	129	3	42	1.7	428	0	No

The Table 1 list includes 4 patients with normal cognition (MSK = 0); five patients that were asymptomatic (MSK = 0.5) and five cognitive impaired patients (MSK = 1). N/A: clinical information not available.

*TMT Label 131 is the reference-pooled material made by adding same protein amount from all specimens of pools 1, 2, and 3 (HIV+ NC, HIV+ A, and HIV+ CI).

^a^Cerebrospinal (CSF) penetration index (CPE).

### M2 proteomics

A total of 3,499 proteins were identified with LC/MS/MS and protein database searching, from which 2,519 proteins, with a minimum of 2 unique peptides, were selected for further analysis. The protein names, accession numbers, sequence coverage, and expectation values are described in S1 Table. Qualitative analysis is described in Table 1. Quantitative analysis is described in S2 Table.

M2 proteomics revealed 28 proteins associated with HACI. There were 9 proteins that were differentially expressed (7 up-regulated and 2 down-regulated) between the HACI categories using p < 0.01 as the statistical significance threshold, and there were 17 proteins that were differentially expressed (15 up and 2 down) using p<0.05 as the statistical significance threshold. We continued to use p<0.05 as statistical significance threshold for our analyses (Table 2). These 17 proteins included enzymes from the glucose metabolic pathways: heat shock proteins; proteins involved in maintenance of cell structure and motility, cell regulation, protein synthesis and turnover, cellular stress and inflammatory response. The impact of a 1.5 fold-change (F.C.) practical significance threshold for the 7 proteins found with this characteristic is described in Table 2.

**Table 2:** List of proteins identified by TMT labeling and differentially expressed among the HACI groups.

Accession	Peptides	Protein Name	NC^1^	A^2^	CI^3^	p value		Biological Process / Molecular Pathway
						NC vs. CI	A vs. CI	
B4DQJ8_HUMAN	8	6-phosphogluconate dehydrogenase, decarboxylating	0.493	0.823	1.528	0.0035		Oxidative stress
B7TY16_HUMAN	2	Actinin alpha 1 isoform 3	0.586	0.784	1.713	0.0091		Cell structure & movement
B2R9S4_HUMAN	5	Capping protein (actin filament), gelsolin-like (CAPG)	0.27	0.59	0.607	0.0063		Cell structure & movement
B4DL49_HUMAN	2	Cathepsin B	0.465	0.431	0.919		0.0315	Neurotoxicity
Q6IPN6_HUMAN	13	Elongation factor 1-alpha	0.3	0.751	0.522	0.0408	0.0294	Protein synthesis
Q5HY54_HUMAN	9	Filamin-A	0.405	0.823	0.945	0.0013		Cell structure & movement
J3KPS3_HUMAN	4	Fructose-bisphosphate aldolase	0.231	0.565	0.765	0.0375		Glycolysis pathway
Q6FGL0_HUMAN	4	Galectin	0.351	0.798	0.846	0.0047	0.0108	Cell regulation
Q2TSD0_HUMAN	17	Glyceraldehyde-3-phosphate dehydrogenase	0.363	0.559	0.848	0.0236		Glycolysis pathway
B3KTV0_HUMAN	7	Heat shock cognate 71 kDa protein	0.379	0.655	0.851	0.0083		Cell protection
Q2VPJ6_HUMAN	4	HSP90AA1 protein	0.319	0.541	1.008	0.008	0.0178	Cell protection
Q53FI1_HUMAN	12	L-plastin variant or Plastin-2	0.419	0.699	1.469	0.0272		Cell structure & movement
Q6PJT4_HUMAN	6	MSN protein (Fragment)	0.41	0.696	0.893	0.0093		Cell structure & movement
B7Z7A9_HUMAN	10	Phosphoglycerate kinase	0.342	0.543	1.1	0.0466		Glycolysis pathway
B4DNK4_HUMAN	21	Pyruvate kinase	0.359	0.64	0.853	0.0165		Glycolysis pathway
B3KPS3_HUMAN	8	Tubulin alpha-ubiquitous chain	0.365	0.53	0.899	0.0091		Cell structure & movement
B0YJC4_HUMAN	7	Vimentin	0.802	1.477	0.788		0.0253	Cell structure & movement

The number of peptides and the mean relative intensities of identified Proteins in MDM from HIV positive women characterized for HACI. 1) NC = normal-cognition; 2) A = Asymptomatic 3) CI = Cognitive Impaired.

### CI vs. NC

**Fig 1:**
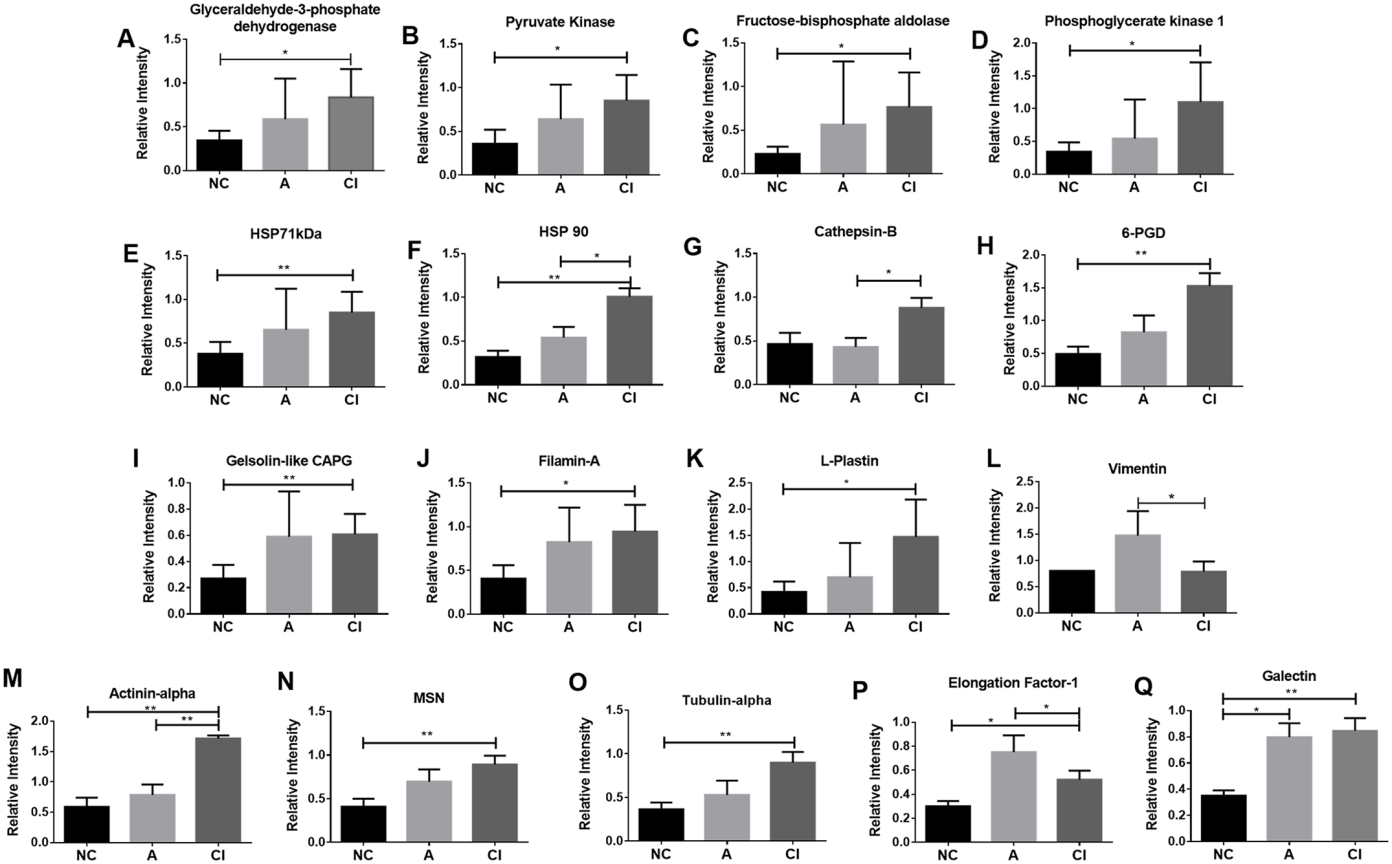
Differentially expressed proteins by TMT and mass spectrometry. A-D are proteins involved in the glycolysis pathway. E and F are the proteins involved with cell protection. G is the protein involved with neurotoxicity. H, and P are the proteins involved in oxidative stress and protein synthesis. From I to O are the proteins involved with the cell structure and motility. *p<0.05; **p<0.01.

Five enzymes related to glucose metabolism were identified as differentially expressed in HACI. These were glyceraldehyde-3-phosphate dehydrogenase (GAPDH), pyruvate kinase (PK), fructose-bisphosphate aldolase (Aldolase), phosphoglycerate kinase 1 (PGK-1), and 6-phosphogluconate dehydrogenase (6PGD) [23–25]. These showed up-regulation with a p <0.05 and a practical significance threshold of +1.5 F.C. in CI vs. NC (Fig 1). Two structural proteins, L-Plastin, Actinin alpha, also showed up-regulation with a p <0.05 and a practical significance threshold of +1.5 F.C. in CI vs. NC (Fig 1). The heat shock proteins HSP71kDa and HSP90AA1 were up-regulated by almost +1.0-F.C. in CI vs. NC. Both proteins are involved in cell protection from stress and the unfolded protein response. The eukaryotic elongation factor-1 (EF-1), a protein involved in cell regulation, was up-regulated by 0.5 F.C. from CI to NC. Gelsolin-like CAPG, filamin-A, moesin (MSN) and tubulin-alpha, proteins involved in cell motility and structure, were up-regulated in CI vs. NC by 0.5 to1.0 F.C.

### A vs. NC and CI

EF-1 was up-regulated in A vs. NC and down-regulated from CI to A by +0.5 F.C. Vimentin was up-regulated in A vs. NC, but it was down-regulated by 1.0 F.C. in CI vs. A. Cathepsin B was not different in A vs. NC, but it was up-regulated in CI vs A with a 0.5 F.C. as shown in Fig 1.

### Ingenuity pathway analyses

**Fig 2:**
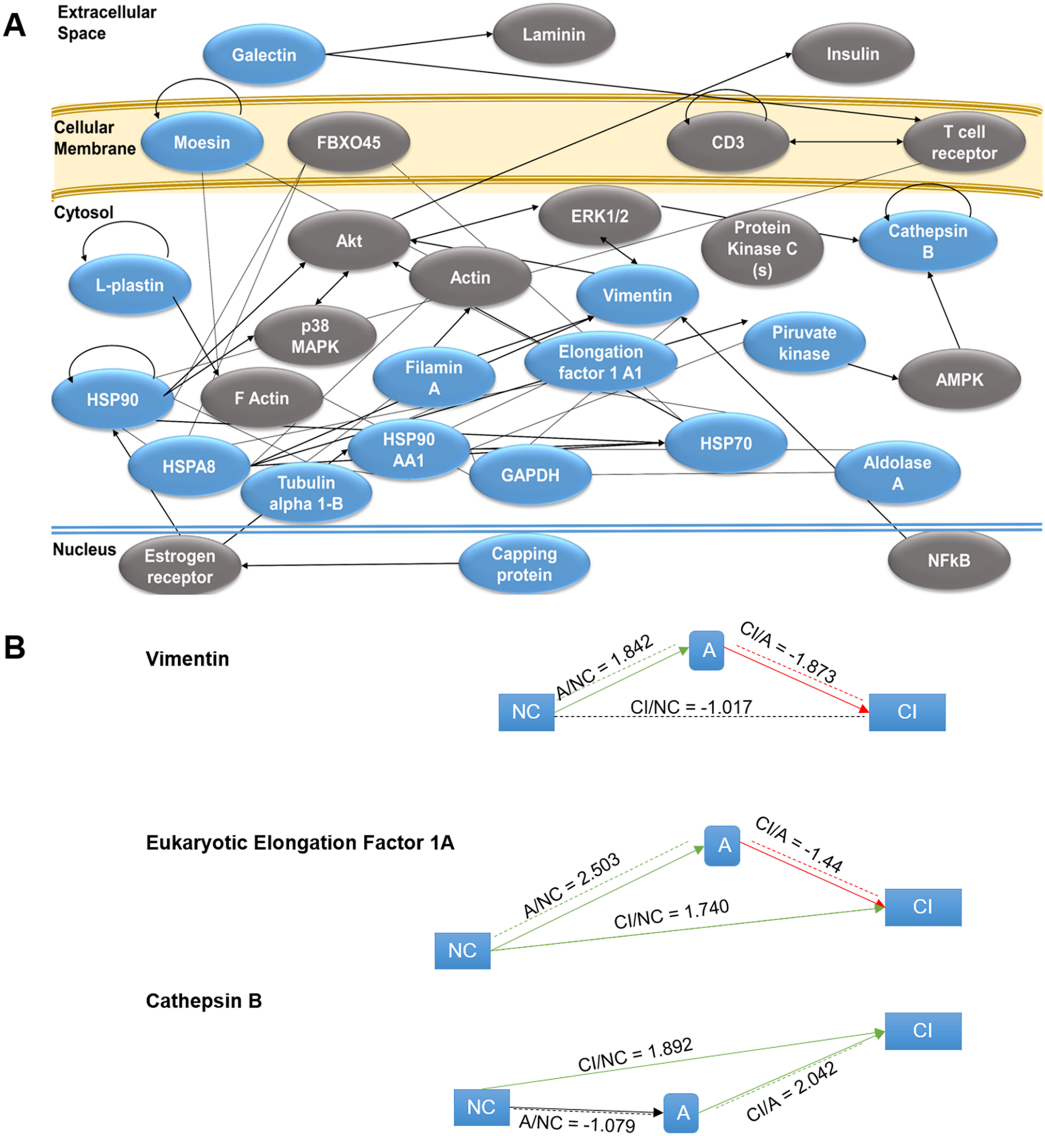
Predicted network of interactions between the proteins identified in macrophages from HACI patients. (A) Blue proteins are from our dataset and the grey colored proteins are the ones that connect the proteins in our dataset according to IPA software. (B) The lower panel shows the pattern of increase/decrease followed by vimentin, EEF1 and cathepsin B among the groups of HACI patients.

The pathway analyses performed with the Ingenuity software for the 17 differentially expressed proteins revealed that most of these proteins can be grouped in canonical pathways associated to glucose metabolism: glycolysis, gluconeogenesis and NADH repair (Fig 2A). The diseases and disorders most associated to the set of proteins analyzed were inflammatory response, immune responses, and neurological diseases. The majority of the proteins have functions related to cellular assembly, organization, function, maintenance and movement. IPA generated a single molecular network grouping all of the proteins, with direct and indirect interactions, associated to cellular assembly and organization, cellular function and maintenance and inflammatory response, for each of the two HACI categories compared to NC: CI vs. NC, A vs. NC and CI vs. A (Fig 2A). Most of the proteins increased in A and CI patients, compared to NC, except for vimentin, EEF-1 and cathepsin B, for which the expression pattern along the groups is depicted in Fig 2B.

### Confirmation of differentially expressed proteins by western blot

**Fig 3:**
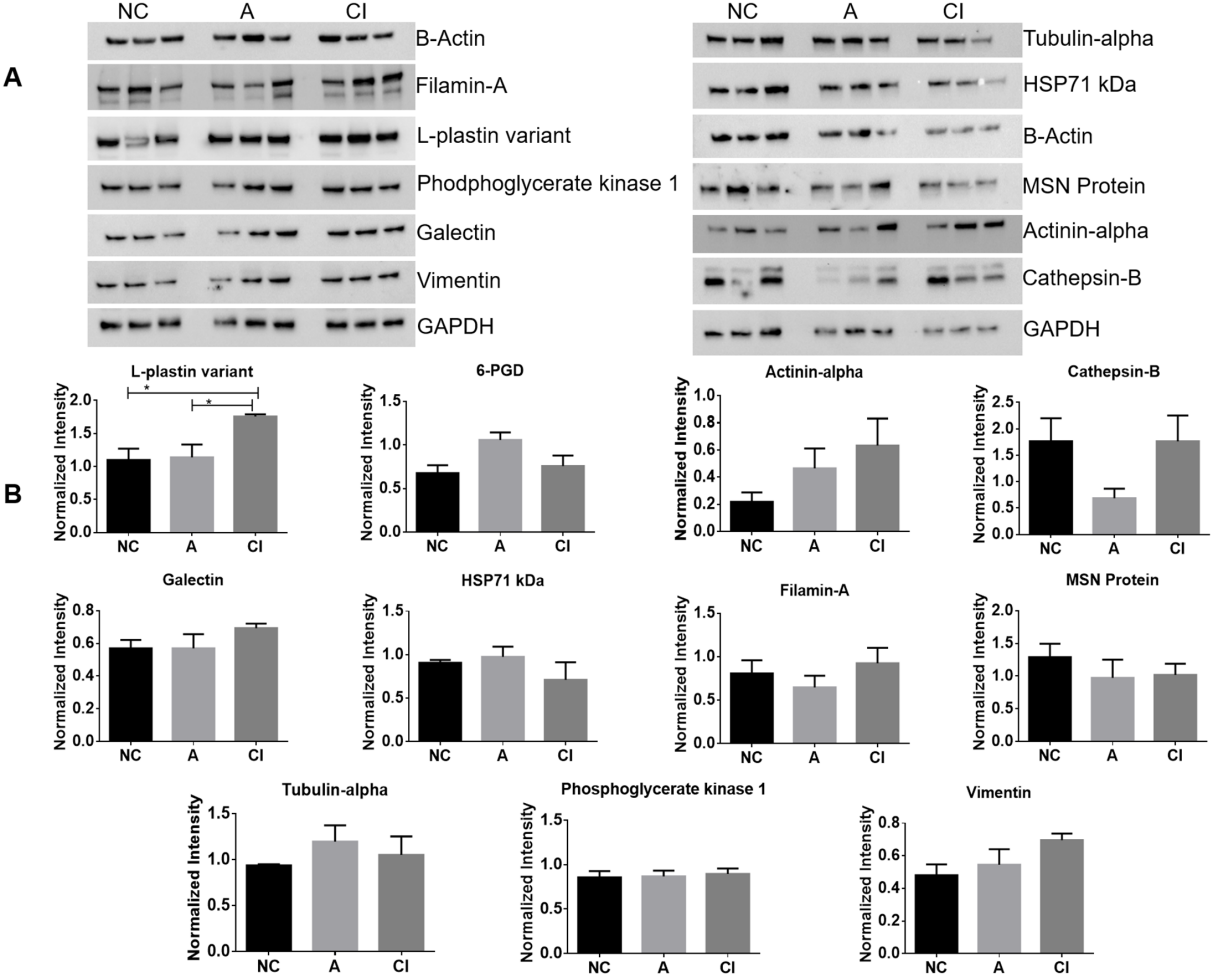
Validation of proteins identified by western blot. (A) Proteins identified by TMT labeling were tested by western blot from MDM lysates of the same patients whose samples were used for proteomics. (B) Densitometry analyses for the western blots were normalized against GAPDH. The statistic analysis between the three groups of patients was performed using One-way ANOVA with a significance of *p<0.05. For Plastin-L, there were significant differences between NC vs CI (p = 0.0316), and between ANI vs CI (p = 0.042).

Western blot was performed with a minimum of three samples from each group of patients (patients 019, 059, 119 and 128 with NC; patients 056, 106, 180 and 181 with A; and patients 001, 166, 172 and 179 with CI). Of the 4 proteins that showed significant differences in CI vs. NC with M2 proteomics, L-Plastin showed significant increased expression in CI vs. NC and in CI vs. A (p = 0.0316 and p = 0.0421, respectively), while 6-PGD exhibited a tendency to increase in A vs. NC, (p = 0.0593) (Fig 3).

To determine if L-Plastin was specific to HIV, five HIV seronegative donors were included in the western blots analyses. Results show a significant increase in L-Plastin in A and CI over HIV seronegative controls (p<0.0001), and HIV seropositive with normal cognition (p<0.0001) (S1 Fig). We did not observe significant differences between HIV seronegatives and HIV seropositives with normal cognition (p = 0.089), supporting a strong association of L-Plastin with cognitive impairment (S1 Fig).

## Discussion

Complementary therapies to prevent the development HACI need to be discovered. As the HIV-infected MDM are the cells that migrate from the blood to the brain inducing inflammation and neurotoxicity, our study was designed to compare the proteins in MDM isolated from patients with HACI to those with normal cognition using quantitative proteomics. Using 14 HIV seropositive patients, proteomics analyses identified a total of 17 proteins differentially expressed, with a significant increase of 7 proteins in patients with HACI: 5 enzymes from the glucose metabolic pathways, and 2 proteins involved in cell structure and motility. However, validation of these proteins by western blots with commercially available antibodies detected significant changes in only two of the 7 proteins detected by proteomics: L-Plastin and 6-PGD. This is not surprising as antibodies detect specific epitopes that may not be the same as those peptides identified by mass spectrometry. Ideal validation is costly as will require custom-made antibodies against the peptides identified by proteomics. It is very important to emphasize that although TMT proteomics is a powerful method to identify and quantify proteins in health and disease, a small proportion of the proteins identified can be validated by western blots as shown in other studies [26,27].

L-Plastin found to be significantly increased with HACI, is a protein involved in controlling the polarization and migration of chemokine-stimulated T-lymphocytes [28]. This protein has been previously identified and validated in the secretome of *in vitro* HIV infected macrophages [29]. These results suggest that HIV alters the expression and secretion of L-Plastin by macrophages to attract T-cells, increasing its efficiency of replication [29]. The 6-PGD enzyme that showed a tendency to increase in A over N group is important in the pentose phosphate pathway (PPP). It converts 6-phospho d-gluconolactone (glucose) to d-ribulose 5-phosphate resulting in the formation of nicotinamide adenine dinucleotide phosphate (NADPH) [30]. NADPH is the molecule required for redox reaction in the lipid production process and cellular oxidative stress, processes by which 6-PGD is associated with cancer and Alzheimer’s disease [31]. In Alzheimer’s disease, glucose-6-phosphate dehydrogenase (G6PD) and 6-PGD both increase in the cortex [30], suggesting that altered glucose metabolism is associated to the development of neurodegenerative disorders. A previous proteomics study assessed the HIV-1 Viral protein (Vpr)-induced macrophage proteome differential expression *in vitro*, also identified glycolytic enzymes that increased with Vpr expression, specifically hexokinase (HK), G6PD, pyruvate kinase M2 (PKM2), and fumarate hydratase (fumarase) [32]. Upregulation of 6-PGD found in A patients suggests the activation of the PPP, which also promotes the synthesis of nucleotide pool for HIV-1 biosynthesis [32]. In another study using this Hispanic Latino women cohort, receptors related to glucose uptake, soluble insulin receptor (sIR) and soluble insulin-like growth factor-1 receptor (sIGF1-R), have been found to correlate with HACI [33]. The sIR and sIGF1-R have crucial roles in glucose metabolism, and their levels in plasma could posit an association with asymptomatic glucose disorders in HIV-seropositive women, which may also lead to the development of HACI [33]. Moreover, a longitudinal study analyzing CSF from HACI patients revealed that alterations in energy metabolites in the CSF might be related to the worsening or improvement in neurocognitive impairment during HIV infection [34].

Excessive protein upregulation can be harmful if it ends in protein aggregation in the brain. It has been shown that some neurodegenerative disorders might be caused by abnormal intra- and/or extracellular deposition of misfolded, aggregated, or ubiquitinated proteins in the brain such as: heat shock proteins, cystatins, cathepsins, tau, and amyloid beta peptides, that result in neuronal dysfunction [35–53]. Amyloid peptides, tau, cathepsin B, and cystatins B and C have been identified in Hispanic Latino cohort of patients with HACI [54–63]. However, western blots did not confirm significant differences for these proteins identified as significant by TMT labeling in the current study: actinin-alpha, cathepsin-B, galectin, HSP70, filamin A, MSN protein, aldolase, tubulin-alpha, PGK-1, and vimentin. One reason for the discrepancy between western blots and M2 proteomics is that the protein quantification via western blot relies on a single signal: the intensity of the expected band on the blot [64], which in turn depends on the specificity of the antibody used and the epitope targeted. Multiple antibodies against different epitopes in the same protein need to be tested because not all antibodies available were monoclonal that could identify the exact epitope detected by proteomics. Moreover, post-translational modifications (PTM) affect the binding of the antibody to its target, and this can be addressed using more than a single antibody to validate one protein by western blot. In contrast, specific peptides and PTMs are identified with LC/MS/MS and protein database searching by retention time, and the mass-to-charge ratio and intensities of precursor and product ions. From this particular experiment, we do not have information about the PTMs of the proteins identified. However, multiplexed, targeted proteomics experiments can be performed for large-scale, quantitative analysis of many peptides and PTMs in a single analysis projects [65], including M2 immunoproteomics methods that leverage antibody-based enrichment of low abundance species [4,14–18].

The major functions affected in HACI are energy production and cellular homeostasis maintenance, probably because the cell needs to increase its processes rates to fight the viral infection, while the virus itself is hijacking the cell to facilitate its replication, viral particles assembly and cell-to-cell transmission. In macrophages, HIV buds in vesicles, therefore it affects the expression of structural proteins [66]. Since there is oxidative and cellular stress during viral infections, the heat shock proteins are also expected to be altered during the infection, in the cells’ effort to protect themselves from apoptosis [67–71]. Altered cellular homeostasis, oxidative stress, and protein synthesis might explain why we have observed lysosomal disruption along with cathepsin B increased secretion from HIV-infected MDM *in vitro* [72–78].

We studied macrophages from 14 patients, 10 of them taking cART. Three of the four patients not taking cART were in the A category, and one in the CI category. This CI patient was virally suppressed and has been under cART regimen in previous cohort visits, with a constant CI diagnosis. We cannot make comparisons on the role of these proteins in cART because most of the patients were treated and the group is small. However, no differences were found between age, plasma viral load, CD4+ cell count or CPE among the three HACI categories. Moreover, 7/14 patients were virally suppressed and in particular 60% of the A and CI patients were suppressed. Therefore, the differences observed in the macrophage protein profile were not related to uneven distribution of clinical parameters between the groups, despite the expected clinical variability. In addition, 16 of the 17 proteins analyzed were significantly higher in CI patients compared to NC. Since both groups, 88% (8 of 9 total) patients were under cART, the increased of glycolysis related proteins found in these patients, appears to be related to cognitive impairment and not to antiviral treatment.

Four of the patients’ samples analyzed were acquired during their first visit to the clinic: three A patients and one CI patient, limiting the possibility of discussing the relation of MDM protein profile to the progression of cognitive function, further limited by the small sample size. We recognize that our findings warrant further longitudinal and mechanistic studies, with a higher number of samples. Unfortunately, we do not have available MDM samples from additional Hispanic Cohort patients per group for proteomics studies, However, we recognize that our findings are relevant and warrant further studies. In addition, since our cohort is entirely comprised of women, this study should be expanded to consider men, since gender differences may result in macrophage proteome differences during HIV-1 infection.

We conclude that L-Plastin and 6-PGD, proteins with different functions in the cell, are associated with HACI. Since they were increased in A and CI patients when compared to NC and HIV seronegative controls, results suggest that the macrophages of patients with moderate to advanced neurocognitive impairment may be undergoing a rate of energy production and cytoskeleton rearrangement different from patients with normal cognition. These proteins are potential candidates for further molecular and longitudinal studies with increased number of patients to ascertain their role in HACI development and to uncover novel targets for therapy.

## Supporting information

S1 Fig Validation of L-Plastin in HIV seronegative controls. (A) L-Plastin was tested by western blot from MDM lysates from HIV-seronegative controls (n = 4) and patients with HACI. (B) Densitometry analyses for the western blots were normalized against GAPDH. The statistical analysis between the three groups of patients was performed using One-way ANOVA with a significance of *p<0.05. For Plastin-L, there were significant differences between C and A (p<0.0001); C and CI (p<0.0001); and NC vs CI (p<0.0001).
(DOCX)

S1 Table Raw data of proteins identified by microwave & Magnetic Proteomics of macrophages from HACI patients. (XLSX)

S2 Table Proteomics analyses of macrophage secretome from HACI patients. (XLSX)

S3 Table Statistics of relative intensities obtained by microwave & magnetic proteomics of macrophages from HACI patients. (DOCX)
