# Supplementary material for: Microwave & magnetic proteomics of macrophages from patients with HIV-associated cognitive impairment
Source: PLoS One. 2017 Jul 26;12(7):e0181779. doi: 10.1371/journal.pone.0181779 (PMC5528838; doi:10.1371/journal.pone.0181779)

**S1 Fig. Validation of L-Plastin in HIV seronegative controls.** (A) L-Plastin was tested by western blot from MDM lysates from HIV-seronegative controls (n=4) and patients with HACI. (B) Densitometry analyses for the western blots were normalized against GAPDH. The statistical analysis between the three groups of patients was performed using One-way ANOVA with a significance of *p<0.05. For Plastin-L, there were significant differences between C and A (p<0.0001); C and CI (p<0.0001); and NC vs CI (p<0.0001).


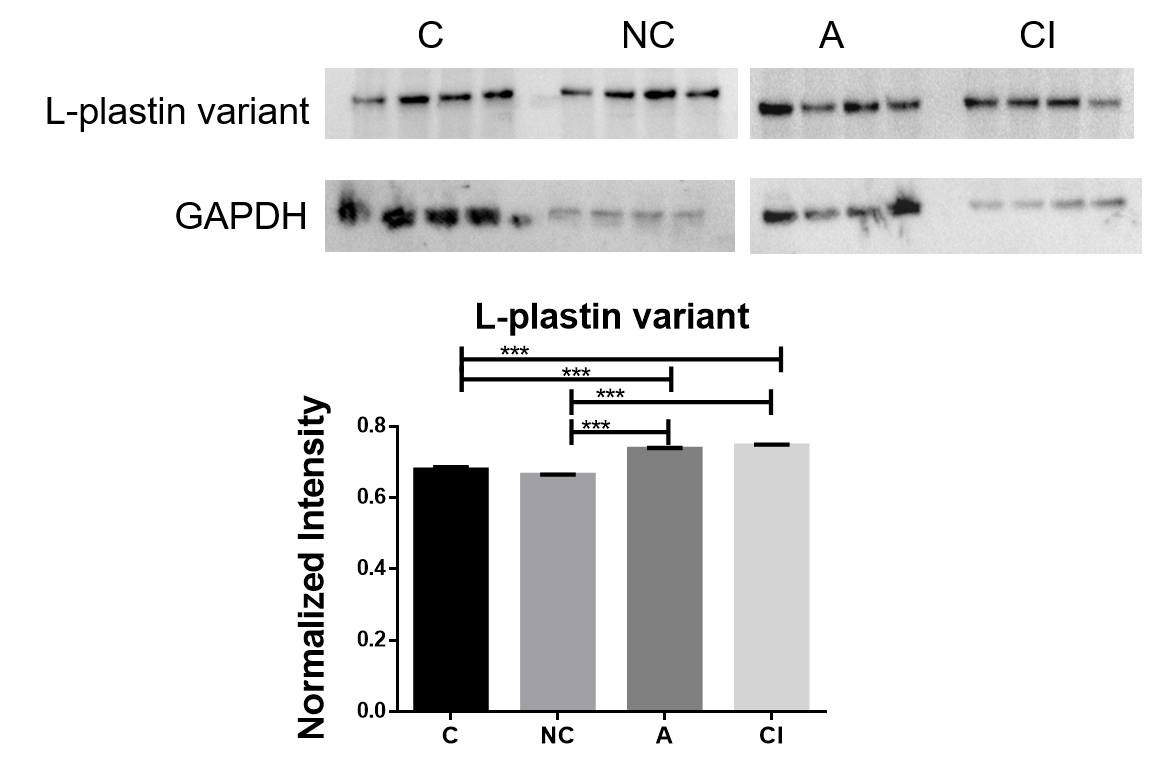

Supplement: S1 Fig — (A) L-Plastin was tested by western blot from MDM lysates from HIV-seronegative controls (n = 4) and patients with HACI. (B) Densitometry analyses for the western blots were normalized against GAPDH. The statistical analysis between the three groups of patients was performed using One-way ANOVA with a significance of *p<0.05. For Plastin-L, there were significant differences between C and A (p<0.0001); C and CI (p<0.0001); and NC vs CI (p<0.0001). (DOCX) [file pone.0181779.s001.docx]
