# Supplementary material for: Microwave & magnetic proteomics of macrophages from patients with HIV-associated cognitive impairment
Source: PLoS One. 2017 Jul 26;12(7):e0181779. doi: 10.1371/journal.pone.0181779 (PMC5528838; doi:10.1371/journal.pone.0181779)
Supplement: S3 Table — (DOCX) [file pone.0181779.s004.docx]

| Average relative intensities of identified proteins | | | | | | | |  | Average relative intensities of identified proteins | | | | | | | |
| --- | --- | --- | --- | --- | --- | --- | --- | --- | --- | --- | --- | --- | --- | --- | --- | --- |
| group | Accession | N  Obs | N | Mean | Std Dev | Min | Max |  | group | Accession | N  Obs | N | Mean | Std Dev | Min | Max |
| A | A4UCS8_HUMAN | 5 | 5 | 0.798 | 1.157 | 0.212 | 2.864 |  | CI | A4UCS8_HUMAN | 5 | 5 | 0.788 | 0.480 | 0.384 | 1.532 |
|  | B0YJC4_HUMAN | 5 | 5 | 1.477 | 0.462 | 1.133 | 2.265 |  |  | B0YJC4_HUMAN | 5 | 5 | 0.788 | 0.191 | 0.514 | 1.032 |
|  | B2R4M6_HUMAN | 5 | 5 | 0.446 | 0.163 | 0.240 | 0.676 |  |  | B2R4M6_HUMAN | 5 | 5 | 0.327 | 0.162 | 0.192 | 0.580 |
|  | B2R4P2_HUMAN | 5 | 5 | 0.344 | 0.325 | 0.177 | 0.924 |  |  | B2R4P2_HUMAN | 5 | 5 | 0.325 | 0.087 | 0.248 | 0.461 |
|  | B2R5H0_HUMAN | 5 | 5 | 0.395 | 0.405 | 0.099 | 1.103 |  |  | B2R5H0_HUMAN | 5 | 5 | 0.345 | 0.224 | 0.150 | 0.705 |
|  | B2R9S4_HUMAN | 5 | 5 | 0.590 | 0.345 | 0.332 | 1.177 |  |  | B2R9S4_HUMAN | 5 | 5 | 0.607 | 0.156 | 0.424 | 0.829 |
|  | B3KPS3_HUMAN | 5 | 5 | 0.530 | 0.366 | 0.253 | 1.168 |  |  | B3KPS3_HUMAN | 5 | 5 | 0.899 | 0.274 | 0.632 | 1.298 |
|  | B3KTV0_HUMAN | 5 | 5 | 0.655 | 0.468 | 0.307 | 1.469 |  |  | B3KTV0_HUMAN | 5 | 5 | 0.851 | 0.237 | 0.593 | 1.152 |
|  | B4DL49_HUMAN | 5 | 5 | 0.431 | 0.232 | 0.273 | 0.832 |  |  | B4DL49_HUMAN | 4 | 4 | 0.880 | 0.251 | 0.643 | 1.118 |
|  | B4DNH8_HUMAN | 5 | 5 | 0.614 | 0.716 | 0.160 | 1.880 |  |  | B4DNH8_HUMAN | 5 | 5 | 1.252 | 0.743 | 0.461 | 2.075 |
|  | B4DNK4_HUMAN | 5 | 5 | 0.640 | 0.396 | 0.334 | 1.315 |  |  | B4DNK4_HUMAN | 5 | 5 | 0.853 | 0.292 | 0.551 | 1.225 |
|  | B4DQJ8_HUMAN | 5 | 5 | 0.823 | 0.573 | 0.248 | 1.570 |  |  | B4DQJ8_HUMAN | 5 | 5 | 1.528 | 0.439 | 0.870 | 1.922 |
|  | B4DRV9_HUMAN | 5 | 5 | 0.587 | 0.465 | 0.210 | 1.399 |  |  | B4DRV9_HUMAN | 5 | 5 | 0.837 | 0.322 | 0.554 | 1.304 |
|  | B7TY16_HUMAN | 5 | 5 | 0.784 | 0.382 | 0.395 | 1.328 |  |  | B7TY16_HUMAN | 5 | 5 | 1.713 | 0.108 | 1.540 | 1.824 |
|  | B7Z7A9_HUMAN | 5 | 5 | 0.543 | 0.597 | 0.189 | 1.607 |  |  | B7Z7A9_HUMAN | 5 | 5 | 1.100 | 0.605 | 0.537 | 1.800 |
|  | D0PNI1_HUMAN | 5 | 5 | 0.637 | 0.795 | 0.198 | 2.056 |  |  | D0PNI1_HUMAN | 5 | 5 | 1.155 | 0.807 | 0.453 | 2.221 |
|  | E2DRY6_HUMAN | 5 | 5 | 0.739 | 1.151 | 0.166 | 2.796 |  |  | E2DRY6_HUMAN | 5 | 5 | 0.735 | 0.419 | 0.380 | 1.394 |
|  | E7ENQ5_HUMAN | 5 | 5 | 0.518 | 0.782 | 0.115 | 1.914 |  |  | E7ENQ5_HUMAN | 5 | 5 | 1.090 | 0.814 | 0.289 | 2.115 |
|  | E9PK25_HUMAN | 5 | 5 | 0.591 | 0.522 | 0.271 | 1.515 |  |  | E9PK25_HUMAN | 5 | 5 | 0.793 | 0.184 | 0.589 | 1.045 |
|  | F2Z393_HUMAN | 5 | 5 | 0.616 | 0.643 | 0.276 | 1.765 |  |  | F2Z393_HUMAN | 5 | 5 | 0.874 | 0.483 | 0.376 | 1.444 |
|  | H7C469_HUMAN | 5 | 5 | 0.346 | 0.286 | 0.175 | 0.854 |  |  | H7C469_HUMAN | 5 | 5 | 0.408 | 0.091 | 0.265 | 0.497 |
|  | J3KPS3_HUMAN | 5 | 5 | 0.565 | 0.722 | 0.154 | 1.851 |  |  | J3KPS3_HUMAN | 5 | 5 | 0.765 | 0.396 | 0.377 | 1.311 |
|  | Q2TSD0_HUMAN | 5 | 5 | 0.559 | 0.428 | 0.229 | 1.309 |  |  | Q2TSD0_HUMAN | 5 | 5 | 0.848 | 0.329 | 0.542 | 1.307 |
|  | Q2TU34_HUMAN | 5 | 5 | 0.728 | 0.946 | 0.175 | 2.396 |  |  | Q2TU34_HUMAN | 5 | 5 | 0.782 | 0.713 | 0.221 | 1.973 |
|  | Q2VPJ6_HUMAN | 5 | 5 | 0.541 | 0.272 | 0.274 | 0.984 |  |  | Q2VPJ6_HUMAN | 5 | 5 | 1.008 | 0.216 | 0.806 | 1.313 |
|  | Q53FI1_HUMAN | 5 | 5 | 0.699 | 0.654 | 0.257 | 1.836 |  |  | Q53FI1_HUMAN | 5 | 5 | 1.469 | 0.712 | 0.681 | 2.216 |
|  | Q53HE2_HUMAN | 5 | 5 | 0.607 | 0.732 | 0.194 | 1.910 |  |  | Q53HE2_HUMAN | 5 | 5 | 0.844 | 0.514 | 0.347 | 1.385 |
|  | Q5HY54_HUMAN | 5 | 5 | 0.823 | 0.395 | 0.432 | 1.449 |  |  | Q5HY54_HUMAN | 5 | 5 | 0.945 | 0.305 | 0.567 | 1.283 |
|  | Q5TZZ9_HUMAN | 5 | 5 | 0.608 | 0.961 | 0.140 | 2.326 |  |  | Q5TZZ9_HUMAN | 5 | 5 | 1.140 | 1.082 | 0.291 | 2.838 |
|  | Q6FGL0_HUMAN | 5 | 5 | 0.798 | 0.239 | 0.593 | 1.062 |  |  | Q6FGL0_HUMAN | 5 | 5 | 0.846 | 0.219 | 0.516 | 1.124 |
|  | Q6IPN6_HUMAN | 5 | 5 | 0.751 | 0.313 | 0.451 | 1.205 |  |  | Q6IPN6_HUMAN | 5 | 5 | 0.522 | 0.167 | 0.381 | 0.747 |
|  | Q6PJT4_HUMAN | 5 | 5 | 0.696 | 0.311 | 0.358 | 1.152 |  |  | Q6PJT4_HUMAN | 5 | 5 | 0.893 | 0.228 | 0.621 | 1.192 |

| Average relative intensities of identified proteins | | | | | | | |  |  |  |  |  |  |  |  |  |
| --- | --- | --- | --- | --- | --- | --- | --- | --- | --- | --- | --- | --- | --- | --- | --- | --- |
| group | Accession | N  Obs | N | Mean | Std Dev | Min | Max |  | Obs | Accession | Method | | t | DF | P | Compare |
| NC | A4UCS8_HUMAN | 4 | 4 | 0.356 | 0.135 | 0.203 | 0.506 |  | 4 | Q2VPJ6_HUMAN | Satterthwaite | | 5.71 | 6.8599 | 0.0008 | NC - CI |
|  | B0YJC4_HUMAN | 2 | 1 | 0.802 | . | 0.802 | 0.802 |  | 7 | B7TY16_HUMAN | Satterthwaite | | 7.09 | 3.6153 | 0.0030 | NC - CI |
|  | B2R4M6_HUMAN | 4 | 4 | 0.439 | 0.193 | 0.284 | 0.714 |  | 8 | B4DQJ8_HUMAN | Satterthwaite | | 4.61 | 6.0764 | 0.0035 | NC - CI |
|  | B2R4P2_HUMAN | 4 | 4 | 0.287 | 0.087 | 0.202 | 0.399 |  | 11 | B7TY16_HUMAN | Satterthwaite | | -5.23 | 4.6357 | 0.0042 | A - CI |
|  | B2R5H0_HUMAN | 4 | 4 | 0.260 | 0.167 | 0.132 | 0.506 |  | 12 | Q6FGL0_HUMAN | Satterthwaite | | 4.68 | 5.2766 | 0.0047 | NC - CI |
|  | B2R9S4_HUMAN | 4 | 4 | 0.270 | 0.105 | 0.138 | 0.393 |  | 13 | B2R9S4_HUMAN | Satterthwaite | | 3.88 | 6.8712 | 0.0063 | NC - CI |
|  | B3KPS3_HUMAN | 4 | 4 | 0.365 | 0.156 | 0.150 | 0.520 |  | 15 | B3KTV0_HUMAN | Satterthwaite | | 3.74 | 6.5132 | 0.0083 | NC - CI |
|  | B3KTV0_HUMAN | 4 | 4 | 0.379 | 0.137 | 0.206 | 0.519 |  | 16 | B3KPS3_HUMAN | Satterthwaite | | 3.68 | 6.4713 | 0.0091 | NC - CI |
|  | B4DL49_HUMAN | 4 | 4 | 0.465 | 0.253 | 0.231 | 0.804 |  | 17 | Q6PJT4_HUMAN | Satterthwaite | | 3.56 | 6.9969 | 0.0093 | NC - CI |
|  | B4DNH8_HUMAN | 4 | 4 | 0.533 | 0.634 | 0.150 | 1.478 |  |  |  |  |  |  |  |  |  |
|  | B4DNK4_HUMAN | 4 | 4 | 0.359 | 0.161 | 0.187 | 0.565 |  |  |  |  |  |  |  |  |  |
|  | B4DQJ8_HUMAN | 4 | 4 | 0.493 | 0.218 | 0.250 | 0.762 |  |  |  |  |  |  |  |  |  |
|  | B4DRV9_HUMAN | 4 | 4 | 0.345 | 0.110 | 0.238 | 0.488 |  |  |  |  |  |  |  |  |  |
|  | B7TY16_HUMAN | 4 | 4 | 0.586 | 0.303 | 0.239 | 0.968 |  |  |  |  |  |  |  |  |  |
|  | B7Z7A9_HUMAN | 4 | 4 | 0.342 | 0.142 | 0.199 | 0.508 |  |  |  |  |  |  |  |  |  |
|  | D0PNI1_HUMAN | 4 | 4 | 0.722 | 0.733 | 0.295 | 1.817 |  |  |  |  |  |  |  |  |  |
|  | E2DRY6_HUMAN | 4 | 4 | 0.333 | 0.131 | 0.194 | 0.485 |  |  |  |  |  |  |  |  |  |
|  | E7ENQ5_HUMAN | 4 | 4 | 0.431 | 0.406 | 0.163 | 1.032 |  |  |  |  |  |  |  |  |  |
|  | E9PK25_HUMAN | 4 | 4 | 0.511 | 0.206 | 0.295 | 0.755 |  |  |  |  |  |  |  |  |  |
|  | F2Z393_HUMAN | 4 | 4 | 0.660 | 0.392 | 0.326 | 1.225 |  |  |  |  |  |  |  |  |  |
|  | H7C469_HUMAN | 4 | 4 | 0.290 | 0.175 | 0.178 | 0.551 |  |  |  |  |  |  |  |  |  |
|  | J3KPS3_HUMAN | 4 | 4 | 0.231 | 0.082 | 0.142 | 0.336 |  |  |  |  |  |  |  |  |  |
|  | Q2TSD0_HUMAN | 4 | 4 | 0.363 | 0.116 | 0.251 | 0.526 |  |  |  |  |  |  |  |  |  |
|  | Q2TU34_HUMAN | 4 | 4 | 0.405 | 0.262 | 0.212 | 0.785 |  |  |  |  |  |  |  |  |  |
|  | Q2VPJ6_HUMAN | 4 | 4 | 0.319 | 0.144 | 0.186 | 0.511 |  |  |  |  |  |  |  |  |  |
|  | Q53FI1_HUMAN | 4 | 4 | 0.419 | 0.198 | 0.201 | 0.612 |  |  |  |  |  |  |  |  |  |
|  | Q53HE2_HUMAN | 4 | 4 | 0.445 | 0.288 | 0.243 | 0.861 |  |  |  |  |  |  |  |  |  |
|  | Q5HY54_HUMAN | 4 | 4 | 0.405 | 0.156 | 0.229 | 0.605 |  |  |  |  |  |  |  |  |  |
|  | Q5TZZ9_HUMAN | 4 | 4 | 0.420 | 0.441 | 0.138 | 1.075 |  |  |  |  |  |  |  |  |  |
|  | Q6FGL0_HUMAN | 4 | 4 | 0.351 | 0.081 | 0.233 | 0.414 |  |  |  |  |  |  |  |  |  |
|  | Q6IPN6_HUMAN | 4 | 4 | 0.300 | 0.088 | 0.195 | 0.407 |  |  |  |  |  |  |  |  |  |
|  | Q6PJT4_HUMAN | 4 | 4 | 0.410 | 0.180 | 0.244 | 0.664 |  |  |  |  |  |  |  |  |  |
